# Preresection Obesity Increases the Risk of Hepatobiliary Complications in Short Bowel Syndrome

**DOI:** 10.3390/nu4101358

**Published:** 2012-09-26

**Authors:** Jon S. Thompson, Rebecca A. Weseman, Fedja A. Rochling, Wendy J. Grant, Jean F. Botha, Alan N. Langnas, David F. Mercer

**Affiliations:** 1 The University of Nebraska Medical Center, Nebraska Medical Center, Omaha, NE 68198, USA; Email: Rebecca.weseman@unmc.edu (R.A.W.); frochling@unmc.edu (F.A.R.); wgrant@unmc.edu (W.J.G.); alangnas@unmc.edu (A.N.L.); dmercer@unmc.edu (D.F.M.); 2 University of the Witwatersrand, Johannesburg, 1 Jan Smuts Avenue, Braamfontein 2000, Johannesburg, South Africa; Email: Jeanf.botha@gmail.com

**Keywords:** short bowel syndrome, obesity, hepatobiliary disease

## Abstract

Patients developing the short bowel syndrome (SBS) are at risk for hepatobiliary disease, as are morbidly obese individuals. We hypothesized that morbidly obese SBS individuals would be at increased risk for developing hepatobiliary complications. We reviewed 79 patients with SBS, 53 patients with initial body mass index (BMI) < 35 were controls. Twenty-six patients with initial BMI > 35 were the obese group. Obese patients were more likely to be weaned off parenteral nutrition (PN) (58% *vs.* 21%). Pre-resection BMI was significantly lower in controls (26 *vs.* 41). BMI at 1, 2, and 5 years was decreased in controls but persistently increased in obese patients. Obese patients were more likely to undergo cholecystectomy prior to SBS (42% *vs.* 32%) and after SBS (80% *vs.* 39%, *p* < 0.05). Fatty liver was more frequent in the obese group prior to SBS (23% *vs.* 0%, *p* < 0.05) but was similar to controls after SBS (23% *vs.* 15%). Fibrosis (8% *vs.* 13%) and cirrhosis/portal hypertension (19% *vs.* 21%) were similar in obese and control groups. Overall, end stage liver disease (ESLD) was similar in obese and control groups (19% *vs.* 11%) but was significantly higher in obese patients receiving PN (45% *vs.* 14%, *p* < 0.05). Obese patients developing SBS are at increased risk of developing hepatobiliary complications. ESLD was similar in the two groups overall but occurs more frequently in obese patients maintained on chronic PN.

## 1. Introduction

Patients developing the short bowel syndrome (SBS) are at risk for hepatobiliary complications, especially if receiving parenteral nutrition (PN) [1]. Approximately one third of SBS patients will develop cholelithiasis [2]. Cholestasis and portal inflammation are present in two thirds of intestinal failure patients on PN; extensive portal fibrosis and cirrhosis develop later in 40% of such patients [3]. These complications have generally been attributed to several therapy related factors, including excess calories or lipid infusion, deficiencies of essential fatty acids or other nutrients, lack of enteral intake, toxicity of components of PN and sepsis [3]. However, there is increasing recognition that patient and intestinal failure related factors are also important [4].

Obesity is a low grade systemic inflammatory condition that is frequently associated with insulin resistance, hyperinsulinemia, hypertension, hyperlipidemia and coronary artery disease (the metabolic syndrome) [5]. Morbidly obese individuals (BMI > 35) have a higher incidence of hepatobiliary disease than normal weight individuals [6,7,8]. Cholelithiasis occurs in obese patients 5 times as often as non-obese (25% *vs.* 5%). The risk of liver disease, such as non-alcoholic fatty liver disease, (NAFLD) is also increased. Simple steatosis is present in 70%–90% morbidly obese patients and steatohepatitis in 20%–40% [7,8], 10%–30% of the latter will progress to cirrhosis and a small proportion to liver failure. Furthermore, obesity increases the risk of advanced fibrosis in chronic liver disease such as chronic hepatitis C [9]. Since obese patients who develop SBS remain obese, they may continue to be at risk for obesity related liver disease [10]. We hypothesized that morbidly obese individuals developing SBS would be at increased risk for developing hepatobiliary complications.

## 2. Methods

We retrospectively reviewed 79 adult patients with SBS evaluated at our center between 1990 and 2008. Fitty-three patients with initial body mass index (BMI) < 35 were controls. Twenty-six patients with initial BMI > 35 were the obese group. SBS was defined as a small intestinal remnant <180 cm in length with associated malabsorption. Records were reviewed to determine patient age and gender, underlying cause of resection, presence of other risk factors for liver disease, status of the intestinal remnant and other digestive organs, presence of hepatobiliary disease pre and post resection, and nutritional management and outcome. Type I anatomy is end jejunostomy, type II jejunocolic anastomosis and type III jejuno-ileocolic anastomosis [9]. Diabetes mellitus (DM) was defined as the clinical diagnosis and treatment of type II diabetes. Bacterial overgrowth was documented by intestinal cultures with >10^5^ bacteria/mL. Central line infections were defined as an episode of culture proven infection and antibiotic treatment. Chronic alcohol use was defined as >1 drink/day for >1 year. Chronic parenteral nutrition (PN) was defined as continued requirement for PN > 1 year after developing SBS. Weight parameters were evaluated at 1, 2, and 5 years after SBS. 

Hepatobiliary disease was evaluated by serum liver function tests, radiologic imaging (ultrasound and computed tomography), endoscopic findings (portal hypertension), and histologic evaluation of liver biopsies. Fibrosis was determined histologically. Biopsy was performed in 11 (21%) controls and 5 (19%) obese patients. Fatty liver was a radiographic diagnosis. Cirrhosis/portal hypertension was determined by a combination of clinical findings, radiographic studies and histology. End stage liver disease (ESLD) was defined as severe hyperbilirubinemia (bilirubin > 6 mg/dL), hypoalbuminemia, and cirrhosis. 

Follow up ranged from 12 to 220 months, with mean follow up of 50 months for controls and 56 months for obese SBS patients. Statistical comparisons were made utilizing analysis of variance and chi square tests, as appropriate, with *p* < 0.05 signifying significance. This study was approved by the UNMC Institutional Review Board.

## 3. Results

There were no differences in age, gender, or cause of resection between the two groups(Table 1). The incidence of hepatitis and cirrhosis was similar prior to SBS in both groups. However, radiographic evidence of fatty liver prior to developing SBS was more frequent in the obese group (23% *vs.* 0%, *p* < 0.05).Obese patients were significantly more likely to have diabetes mellitus (23% *vs.* 4%, *p* < 0.05). Incidence of alcohol use, bacterial overgrowth and central line infection was similar (Table 1). Intestinal remnant length, anatomy type, and presence of the colon were not different between the two groups (Table 2).

**Table 1:** Comparison of patient groups.

			BMI < 35	BMI > 35
**Number**			53	26
**Age mean range (years)**			50 (19–80)	53 (31–82)
**Sex**		**Female**	37 (70%)	17 (65%)
		**Male**	16 (30%)	9 (35%)
**Ethnicity **		**Caucasian **	46 (87%)	24 (92%)
		**Hispanic**	4 (5%)	2 (0%)
		**Afro-American**	3 (8%)	0 (8%)
**Diagnosis**				
	**Postoperative SBS**	****	16 (30%)	8 (31%)
	**Mesenteric Vasc. Disease**	****	11 (21%)	7 (27%)
	**Cancer/Irradiation**	****	11 (21%)	3 (12%)
	**Crohns Disease**	****	8 (15%)	3 (12%)
	**Other**	****	6 (11%)	5 (19%)
**Medical history**				
	**Bacterial overgrowth**		16 (29%)	7 (27%)
	**Central line infections**		30 (57%)	16 (62%)
	**Chronic alcohol use**		7 (13%)	2 (8%)
	**Cirrhosis**	****	0 (0%)	0 (0%)
	**Diabetes Mellitus**		2 (4%)	6 (23%) *
	**Fatty liver**		0 (0%)	6 (23%) *
	**Hepatitis**		2 (4%)	0 (0%)

BMI = Body Mass Index (kg/m^2^); SBS = Short Bowel Syndrome; * *p* < 0.05 *vs.* BMI < 35.

**Table 2:** Comparison of intestinal anatomy.

		BMI < 35	BMI > 35
**Number**		53	26
**Intestinal Length (cm)**			
****	**<60**	21 (40%)	9 (35%)
****	**60–120**	18 (34%)	10 (38%)
****	**>120**	14 (26%)	7 (27%)
**Colon Present**		44 (83%)	23 (88%)
**Anatomy type**			
****	**I**	16 (30%)	7 (27%)
****	**II**	29 (55%)	13 (50%)
****	**III**	8 (15%)	6 (23%)

BMI = Body Mass Index (kg/m^2^).

Surgical rehabilitation (ostomy closure, intestinal lengthening and intestinal transplantation) had a similar occurrence in obese and control groups (46% *vs.* 36%) (Table 3). Transplants included 3 liver-small intestine and 3 isolated small intestine in controls and 1 liver-small intestine and 1 isolated small intestine in obese patients. Obese patients were more likely to be weaned off PN within 12 months of developing SBS (58% *vs.* 21%, *p* < 0.05). Obese patients were less likely to require PN at each category of remnant length and intestinal anatomy. Pre-resection BMI in controls was significantly lower than in obese patients. BMI at 1, 2, and 5 years was decreased in both controls and obese patients but was persistently increased in obese patients compared to controls (Table 4).

**Table 3:** Comparison of intestinal rehabilitation.

		BMI < 35	BMI > 35
**Number**		53	26
**Medical rehabilitation**			
****	**No PN**	8 (15%)	7 (27%)
****	**PN <** **1 ** **year**	3 (6%)	8 (31%)
****	**PN >1** **year**	42 (79%)	11 (42%) *
**PN by Remnant length (cm)**			
****	**<60**	20/21 (95%)	5/9 (56%) *
****	**60–120**	13/18 (72%)	3/10 (30%) *
****	**>120**	9/14 (64%)	3/7 (43%)
**PN by Anatomy type**			
****	**I**	12/16 (75%)	4/7 (57%)
****	**II**	24/29 (83%)	4/13 (31%) *
****	**III**	6/8 (75%)	3/6 (50%)
**Surgical rehabilitation**			
****	**Close ostomy**	4	8
****	**Reversed segment**	3	0
	**Intestinal lengthening**	6	2
	**Intestinal transplant**	6	2
****	**Total**	19 (36%)	12 (46%)
****	**Deaths**	4 (8%)	4 (15%)

* *p* < 0.05 *vs.* BMI < 35; BMI = Body Mass Index (kg/m^2^); PN = Parenteral Nutrition.

**Table 4:** Comparison of body mass index.

			BMI < 35	BMI > 35
**Number**			53	26
**BMI (kg/m^2^)**				
****	**Mean**			
****		**Pre SBS**	26 ± 5 ^#^	41 ± 9
****		**1 year**	23 ± 4 *^,#^	34 ± 7 *
****		**2 years**	24 ± 3 *^,#^	34 ± 7 *
****		**5 years**	22 ± 3 *^,#^	33 ± 9 *
****	**No. BMI >** ** 35**			
****		**Pre SBS**	0/53 (0%)	26/26 (100%)
****		**1 year**	0/34 (0%)	8/20 (40%)
****		**2 years**	0/29 (0%)	6/16 (38%)
****		**5 years**	0/25 (0%)	4/16 (25%)
**IBW (%)**				
****		**Pre SBS**	122 ± 20 ^#^	190 ± 43
****		**1 year**	107± 19 *^,#^	156 ± 34 *
****		**2 years**	102 ± 15 *^,#^	160 ± 33 *
****		**5 years**	101 ± 13 *^,#^	154 ± 40 *

* *p* < 0.05 *vs.* Pre SBS; ^#^*vs.* BMI > 35; BMI = Body Mass Index; IBW = Ideal Body Weight.

Obese patients were more likely to have undergone cholecystectomy prior to SBS (42% *vs.* 32%, *p* < 0.05) (Table 5). Obese patients were also more likely to undergo cholecystectomy after developing SBS (80% *vs.* 36%, *p* < 0.05). There was no difference in the incidence of postresection cholecystectomy with or without chronic PN in the control (39% *vs.* 20%) and obese patients (86% *vs.* 75%).

**Table 5:** Biliary disease in short bowel syndrome (SBS) patients.

		BMI < 35			BMI > 35		
		PN	No PN	Total	PN	No PN	Total
**Number**		42	11	53	11	15	26
**Cholecystectomy **							
****	**Pre SBS**	11 (26%)	6 (55%)	17 (32%)	4 (36%)	7 (47%)	11 (42%) *
****	**Post SBS**	12 (29%)	1 (9%)	13 (22%)	6 (55%)	6 (40%)	12 (46%) *
**Normal**							
****	**Gallbladder**	16 (38%)	4 (36%)	20 (38%)	1 (9%)	2 (13%)	3 (12%)
****	**Prophylactic**	3 (7%)	0 (0%)	3 (6%)	0 (0%)	0 (0%)	0 (0%)
**Cholecystectomy**							

* *p* < 0.05 *vs.* BMI < 35; SBS = Short Bowel Syndrome; PN = Parental Nutrition >1 year; BMI = Body Mass Index (kg/m^2^).

Overall, the incidence of ESLD was similar in obese and control groups (19% *vs.* 11%) but was significantly higher in those patients receiving chronic PN (45% *vs.* 14%, *p* < 0.05) (Table 6). Radiographic evidence of fatty liver in the obese group was similar to controls after SBS (23% *vs.* 15%). Fibrosis and cirrhosis/portal hypertension were also similar in obese and control groups.

**Table 6:** Liver disease and its outcome.

		BMI < 35			BMI > 35		
		PN	No PN	Total	PN	No PN	Total
**Number**		42	11	53	11	15	26
**Fatty Liver**		8 (19%)	0 (0%)	8 (15%)	4 (36%)	2 (13%)	6 (23%)
**Fibrosis**		7 (17%)	0 (0%)	7 (13%)	2 (18%)	0 (0%)	2 (8%)
**Cirrhosis/PHT**		11 (26%)	0 (0%)	11 (21%)	4 (36%)	1 (7%)	5 (19%)
**End Stage Liver Disease**							
****	**Alive**	3 (7%)	0 (0%)	3 (6%)	0 (0%)	0 (0%)	0 (0%)
	**Liver Transplant**	3 (7%)	0 (0%)	3 (6%)	1 (9%)	0 (0%)	1 (4%)
	**Dead**	0 (0%)	0 (0%)	0 (0%)	4 (36%)	0 (0%)	4 (15%)
****	**Total**	6 (14%)	0 (0%)	6 (11%)	5 (45%)	0 (0%)	5 (19%)

*p < 0.05 *vs.* BMI < 35; PN = Parenteral Nutrition >1 year; PHT = Portal Hypertension; BMI = Body Mass Index (kg/m^2^).

## 4. Discussion

An increasing number of morbidly obese individuals are developing SBS. This may be related to complications of bariatric procedures or the treatment of other intestinal disease in obese patients [10,11]. However, obesity is not a commonly recognized risk factor for outcome of intestinal failure [12].

Obese patients developing SBS were at increased risk of developing certain hepatobiliary complications compared to non-obese individuals. In the present study they were almost three times more likely to undergo cholecystectomy after developing SBS. The development of ESLD was similar in the two groups overall but occurred more frequently in obese than non-obese patients when maintained on PN for more than one year. The incidence of fatty liver, fibrosis, and cirrhosis was similar in the two groups.

The explanation for these findings may rest in several important differences in the obese and non-obese SBS patients. Many of the obese patients remained morbidly obese after developing SBS. 40% still had a BMI > 35 two years after developing SBS and the mean BMI at five years was 33. Thus, they would remain at risk for obesity related liver disease and other inflammatory complications related to the metabolic syndrome. 

Obese patients were less likely to require long term PN compared to non-obese patients. Overall, this should reduce the risk of complications in this group. However, those obese patients that were receiving PN were at greatest risk for ESLD. In this context PN may present another example of a “second hit” in patients with preexisting steatosis than can lead to the development of steatohepatitis and subsequent fibrosis [7,8]. This suggests that both obesity and PN are important determinants of ESLD in SBS patients. 

Obese patients have a higher incidence of pre-existing hepatobiliary disease. As noted previously, obese SBS patients were more likely to develop biliary tract disease preoperatively [6]. They were significantly more likely to develop cholelithiasis and cholecystitis post resection as well. Thus, the factors increasing risk of cholelithiasis preoperatively may remain after developing SBS. Obese SBS patients also had a higher incidence of preexisting fatty liver. However, the frequency of radiologic fatty liver was similar to nonobese patients after developing SBS. This is an interesting observation that suggests that despite maintaining a higher BMI the obese patients have a reduced tendency to develop hepatic steatosis after developing SBS.

There is a unique gut microbiome in obese individuals which might harvest more energy from the diet and also direct the host response to energy intake [13]. It has been speculated that this might produce the nutritional advantage manifest by preserved body weight and decreased PN requirements reported previously and further substantiated in the present study [11]. However, it is not clear if intestinal resection modifies the gut microbiome and more specifically the obese gut microbiome [14]. The gut microbiome may play a role in the development of nonalcoholic fatty liver disease (NAFLD) [15]. The obesity gut microbiome might also promote other types of liver injury. One potential mechanism might be loss of barrier function, which can occur in SBS patients, and predispose to inflammatory injury in the liver [5]. There is experimental evidence that obesity is associated with increased intestinal permeability and increased portal lipopolysaccharide levels which might contribute to liver inflammatory damage [16]. There was no difference in the incidence of bacterial overgrowth in the two groups in the present study.

Obese SBS patients were more likely to have type 2 diabetes mellitus than their non-obese counterparts. Insulin resistance is a predictor of NAFLD and advanced hepatic fibrosis in obese patients [8,17]. Of the five patients developing ESLD in the obese group, three had BMI > 30 and DM (diabetes mellitus), and two had BMI < 25 and no DM. Thus, it is not clear if obesity and DM are independent risk factors in this small patient sample. 

There are several limitations to the present study. This is a retrospective study and patients were not evaluated in a uniform fashion. Histology was not routinely obtained or available and thus we are unable to perform a more sophisticated analysis of hepatic pathology. This is a referral based group of patients which may not accurately reflect the overall population of obese SBS patients. The incidence of liver disease may be greater in our population. We do not have specific information about dietary intake, e.g., fat or specific PN requirements. Reverse causation is another potentially confounding factor since some of the patients with normal weight at the time of SBS may have been obese previously. However, as this is the largest group of obese SBS patients reported in the in the literature, the observations made should have significance.

## 5. Conclusions

In summary, obesity is an important factor in the outcome of SBS. Obese patients maintain a higher BMI and are less likely to require long term PN than non-obese individuals. The present study suggests that obese patients developing SBS are at increased risk for hepatobiliary complications, especially if receiving PN.

## References

[1] Cavicchi M., Beau P., Crenn P., Degott C., Messing B. (2000). Prevalence of liver disease and contributing factors in patients receiving home parenteral nutrition for permanent intestinal failure. Ann. Int. Med..

[2] Thompson J.S. (1996). The role of prophylactic cholecystectomy in the short bowel syndrome. Arch. Surg..

[3] Chan S., McCowen K.C., Bistrian B.R., Thibault A., Keane-Ellison M., Forse R.A., Babineau T., Burke P. (1999). Incidence, prognosis and etiology of end-stage liver disease in patients receiving home parenteral nutrition. Surgery.

[4] Kelly D. (2006). Intestinal failure associated liver disease: What do we know today?. Gastroenterology.

[5] Iyer A., Fairlie D.P., Prins J.B., Hammock B.D., Brown L. (2010). Inflammatory lipid mediators in adipocyte function and obesity. Nat. Rev. Endocrinol..

[6] Dittrick G.W., Thompson J.S., Campos D., Bremers D., Sudan D. (2005). Gallbladder pathology in morbid obesity. Obes. Surg..

[7] Frantzides C.T., Carlson M.A., Moore R.E., Zografakis J.G., Madan A.K., Puumala S., Keshavarzian A. (2004). Effect of body mass index on nonalcoholic fatty liver disease in patients undergoing minimally invasive bariatric surgery. J. Gastrointest. Surg..

[8] Mathurin P., Hollebecque A., Arnalsteen L., Buob D., Leteurtre E., Caiazzo R., Pigeyre M., Verkindt H., Dharancy S., Louvet A. (2009). Prospective study of the long term effects of bariatric surgery in liver injury in patients without advanced disease. Gastroenterology.

[9] Carbonnel F., Cosnes J., Chevret S., Beaugerie L., Ngo Y., Malafosse M., Parc R., Le Quintrec Y., Gendre J.P. (1996). The role of anatomic factors in nutritional autonomy after extensive small bowel resection. JPEN J. Parenter Enteral. Nutr..

[10] Riley T.R., Taheri M., Scherbman J.R. (2009). Does weight loss history affect fibrosis in the setting of chronic liver disease?. J. Gastrointest. Liver Dis..

[11] Thompson J.S., Weseman R., Rochling F., Grant W., Botha J., Langnas A., Mercer D. Pre-Resection BMI Influences Postresection BMI in Short Bowel Syndrome. Proceedings of the XI International Small Bowel Transplant Symposium.

[12] Beath S., Pironi L., Gabe S., Horslen S., Sudan D., Mazeriegos G., Steiger E., Goulet O., Fryer J. (2008). Collaborative strategies to reduce mortality and morbidity in patients with chronic intestinal failure including those who are referred for small bowel transplantation. Transplantation.

[13] Tilg H., Moschen A.R., Kaser A. (2009). Obesity and the microbiota. Gastroenterology.

[14] Hartman A.L., Lough D.M., Barupal D.K., Fiehn O., Fishbein T., Zasloff M., Eisen J.A. (2009). Human gut microbiome adopts an alternative state following small bowel transplantation. Proc. Natl. Acad. Sci..

[15] Son G., Kremer M., Hines I.N. (2010). Contribution of gut bacteria to liver pathobiology. Gastroenterol. Res. Pract..

[16] Brun P., Castagliuolo I., Di Leo V., Buda A., Pinzani M., Palu G., Martines D. (2007). Increased intestinal permeability in obese mice: New evidence in the pathogenesis of nonalcoholic steatohepatitis. Am. J. Physicol. Gastrointest. Liver Physiol..

[17] Beymer C., Kowdlay K.V., Langnas A., Edmonson P., Dellinger E.P., Flum D.R. (2003). Prevalence and predictors of asymptomatic liver disease in patients undergoing gastric bypass surgery. Arch. Surg..

